# Chimerization Enables Gene Synthesis and Lentiviral Delivery of Customizable TALE-Based Effectors

**DOI:** 10.3390/ijms21030795

**Published:** 2020-01-25

**Authors:** Yongxing Fang, Wladislaw Stroukov, Toni Cathomen, Claudio Mussolino

**Affiliations:** 1 Institute for Transfusion Medicine and Gene Therapy, Medical Center—University of Freiburg, 79106 Freiburg, Germany; yongxing.fang@uniklinik-freiburg.de (Y.F.); wladislaw.stroukov@kcl.ac.uk (W.S.); toni.cathomen@uniklinik-freiburg.de (T.C.); 2 Center for Chronic Immunodeficiency (CCI), Medical Center—University of Freiburg, 79106 Freiburg, Germany; 3 Faculty of Medicine, University of Freiburg, 79106 Freiburg, Germany

**Keywords:** delivery of epigenome editors, gene therapy, transcription activator-like effectors

## Abstract

Designer effectors based on the DNA binding domain (DBD) of *Xanthomonas* transcription activator-like effectors (TALEs) are powerful sequence-specific tools with an excellent reputation for their specificity in editing the genome, transcriptome, and more recently the epigenome in multiple cellular systems. However, the repetitive structure of the TALE arrays composing the DBD impedes their generation as gene synthesis product and prevents the delivery of TALE-based genes using lentiviral vectors (LVs), a widely used system for human gene therapy. To overcome these limitations, we aimed at chimerizing the DNA sequence encoding for the TALE-DBDs by introducing sufficient diversity to facilitate both their gene synthesis and enable their lentiviral delivery. To this end, we replaced three out of 17 *Xanthomonas* TALE repeats with TALE-like units from the bacterium *Burkholderia rhizoxinica*. This was combined with extensive codon variation and specific amino acid substitutions throughout the DBD in order to maximize intra- and inter-repeat sequence variability. We demonstrate that chimerized TALEs can be easily generated using conventional Golden Gate cloning strategy or gene synthesis. Moreover, chimerization enabled the delivery of TALE-based designer nucleases, transcriptome and epigenome editors using lentiviral vectors. When delivered as plasmid DNA, chimerized TALEs targeting the *CCR5* and *CXCR4* loci showed comparable activities in human cells. However, lentiviral delivery of TALE-based transcriptional activators was only successful in the chimerized form. Similarly, delivery of a chimerized *CXCR4*-specific epigenome editor resulted in rapid silencing of endogenous *CXCR4* expression. In conclusion, extensive codon variation and chimerization of TALE-based DBDs enables both the simplified generation and the lentiviral delivery of designer TALEs, and therefore facilitates the clinical application of these tools to precisely edit the genome, transcriptome and epigenome.

## 1. Introduction

The availability of platforms capable of binding to predefined sites in the human genome enables the targeted activity of designer effectors at predefined sites. In the genome editing field, the fusion of endonuclease domains to customizable DNA binding domains (DBDs) has been widely used to introduce targeted DNA double strand breaks in defined genomic regions [1]. This, in turn, activates the cellular DNA repair pathways, namely non-homologous end-joining or homology directed repair, which can be harnessed either to inactivate genes or to achieve targeted sequence changes, respectively [2]. Similarly, customizable DNA binding domains have been explored to alter the transcriptome of a cell through their fusion with transcriptional activator or repressor domains [3]. In recent years, a similar approach has enabled targeted changes in the epigenome using designer effectors capable of introducing alterations of the cellular epigenome in a precise manner [4,5]. Both transcriptome and epigenome editors can be targeted to gene promoters or enhancers, thus, allowing sustainable control of gene expression in a targeted fashion. A commonality of the different approaches described is the presence of a DNA binding domain that defines the genomic target where the effector function is exploited. Among others, the DNA binding domains derived from transcription activator-like effectors (TALE) of *Xanthomonas* bacteria have been extensively used, in the last decade, for their high specificity and simplicity for generating novel DBDs [6], and therefore, to date, representing one of the most successful tools to target specific sequences in the large mammalian genome. The strength of this DNA binding domain lies in its structure, i.e., a variable length array of 15.5 to 17.5 modules each one capable of binding to a single nucleotide of the target sequence. Each module is composed of 34 amino acids except for the last “half” module which is typically shorter [7]. The specificity of DNA binding is dictated by the two amino acids in position 12 and 13 within each module, the repeat-variable diresidue or RVD, and is based on a simple ‘single nucleotide to one RVD’ code, that has revolutionized the field of targeted DNA modifications in the last decade [8]. Since the DNA binding of each module is independent of the neighboring unit, the four different repeat modules, each targeting one of the four bases, can be assembled in an array inferred by the DNA target sequence [9,10]. While this simplifies the availability of customized DNA binding domains and has represented a major breakthrough in the last decades [11], the sequence repetitiveness of these conserved modules is a major limitation. Indeed, the modules are virtually identical to each other at the protein level except for the RVDs [7]. This has represented a major limitation for the assembly of new DBDs and makes the generation of TALE-DBDs with novel specificities somewhat challenging. It generally requires the application of complex design strategies based on “Golden Gate cloning” as the highly repetitive structure impedes direct DNA synthesis. This in turn has prevented the widespread use of this technology thus far.

The delivery of effectors harboring a TALE-DBD to the target cells is generally achieved using well-established procedures to transfer plasmid DNA or mRNA encoding for designer effectors to cell lines and primary cells of different origin, respectively [12]. Since these strategies typically promote the expression of the effectors for a limited time (i.e., hours in case of mRNA or a few days when using plasmid DNA) they are particularly suited in so-called hit-and-run approaches. While this is desired when using designer nucleases to avoid cytotoxicity [13], in the context of transcriptome editing the sustained expression of a designer transcription factor is essential [4]. Similarly, transient expression of an epigenome editor is sufficient for sustained epigenome editing in cell lines [14] but might result in the loss of the newly introduced epigenetic mark in cells that undergo differentiation, such as stem cells. In these cases, sustained expression of the designer transcription factor or epigenome editor could contribute to the stability of the desired modification. Recombinant viral vectors such as adenovirus (Ad) or adeno-associated virus (AAV) have been largely used to deliver designer effectors [15]. In contrast, HIV-1-based lentiviral vectors have failed in this endeavor [16,17]. This has been largely attributed to the mechanism of retroviral replication that involves template switching events for the completion of DNA synthesis operated by the viral reverse transcriptase. As a consequence, additional intramolecular switching events during viral replication of highly repetitive genomes could result in loss of the intervening sequences between the repetitive units [16,17]. Given the complex procedures to generate recombinant adenoviral vectors and the limited cargo capacity offered by AAV vectors, the possibility to deliver TALE-based effectors with the widely used lentiviral vector platform would have major advantages for the research community. In previous efforts, in silico evolution strategies have been used to obtain DNA sequences encoding for TALE-DBD devoid of any repeat longer than 12 base pairs [18]. While this has allowed the delivery of functional TALE-based activators using lentiviral vectors, it still retained repeats longer than eight base pairs. Therefore, the DNA sequence of a recoded TALE-DBD (reTALE) cannot be synthesized as gene fragments, a fast and affordable method of gene synthesis widely used nowadays. This in turns limits the adoption of TALE-based effectors by nonspecialized laboratories. To overcome these limitations, we have devised a strategy to further reduce intramolecular repetitiveness of the DNA sequence coding for TALE-based effectors. To this end, we have performed a two-step optimization of the codon usage within each TALE repeat to both maximize the expression in mammalian systems and reduce repeats longer than eight base pairs. To further increase sequence variation, we introduced amino acid substitutions at specific positions that are naturally variable [8]. In addition, we replaced three repeats within a TALE-DBD with corresponding modules from TALEs found in the bacterium *Burkholderia rhizoxinica* that share limited sequence identity with *Xanthomonas* TALEs [19]. When delivered as plasmid DNA, the resulting chimerized TALEs are as efficient as the original effectors in editing the genome when fused to the *Fok*I endonuclease domain or the transcriptome when fused to the VP64 transcription activator, respectively. However, lentiviral delivery of TALE-based transcription activator or epigenome editors is only possible when chimerized TALEs are used. Our newly developed chimerized TALE platform allows the generation of TALE-based effectors in a fast and straightforward manner by gene synthesis. Moreover, vectorization of effectors containing a chimerized TALE-DBD in lentiviral vectors provides new modalities to modify the genome, epigenome, and transcriptome of sensitive primary human cells.

## 2. Results

### 2.1. Chimerization of TALE-Based DNA Binding Domains Results in the Assembly of Functional Effectors

**Figure 1:**
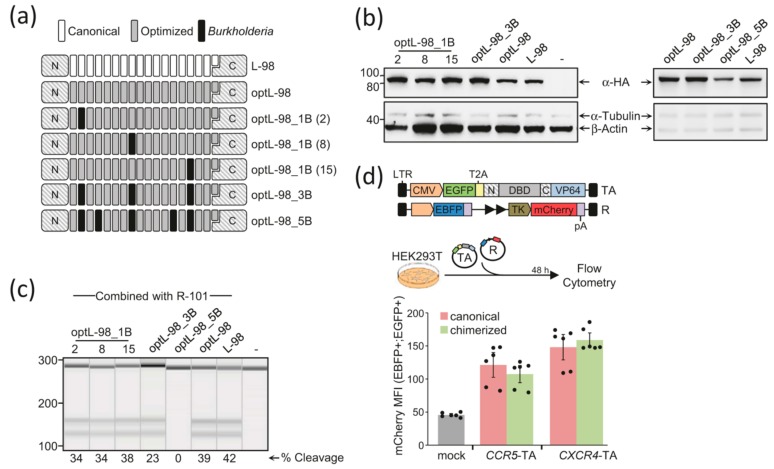
Chimerization results in functional TALE-based nucleases and transcriptional activators. (**a**) Schematics of chimerized TALE DNA binding domains (DBDs). The DNA sequence coding for canonical TALE-DBD (white) is extensively modified to minimize intra- and inter-module repetitiveness (grey). In addition, chimerized TALE-DBD include TALE repeats derived from the bacterium *Burkholderia rhizoxinica* (black). (**b**) Expression levels of chimerized TALE nucleases. Expression levels were determined by immunoblotting using antibodies against HA-tag (top panel). A combination of antibodies recognizing α-Tubulin and β-Actin (lower panel) are used to normalize the total protein content. The positions of the relevant proteins are indicated in the middle and the protein marker (in kDa) on the left. (**c**) Activity of optimized and chimerized TALE nucleases. Capillary electrophoresis gel image (QIAxcel System, Qiagen) showing the target locus disruption assayed by T7 Endonuclease 1 (T7E1) assay. The extent of cleavage (as percentage of modified alleles) is indicated below each lane. (**d**) Activity of chimerized transcriptional activators (TA). To monitor the activity of the TAs, we generated a reporter construct (R) that included the corresponding binding sites of the TAs in tandem (black triangles), followed by a minimal promoter fragment from the HSV thymidine kinase (TK) gene adjacent to a mCherry expression cassette (upper panel). The expression cassette for an enhanced blue fluorescent protein (EBFP) driven by the CMV is included to track the reporter construct. The activity of TAs was measured by co-transfecting the corresponding TALE-TA expression plasmid and the reporter in HEK293T cells (upper panel). The TAs are driven by a cytomegalovirus (CMV) promoter and, to track their expression, are fused to the C-terminus of an enhanced green fluorescent protein (EGFP) via a T2A peptide. TAs containing either the canonical (light red) or the best-performing chimerized TALE-DBD (light green) were compared side-by-side for their ability to bind to their intended target sites and drive mCherry expression via flow cytometry, 48 h post transfection. The histogram shows the mCherry expression levels (mean ± SEM), measured as mean fluorescence intensity (MFI), in the fraction of cells that received both the effector (EGFP+) and the reporter plasmids (EBFP+). Each dot represents a single data point. Basal mCherry expression levels are measured by transfecting an effector plasmid lacking the DNA binding domain (mock, grey bar). pA, poly adenylation signal and LTR, long terminal repeat.

We reasoned that minimizing the repetitiveness of the DNA sequence coding for a TALE-DBD would allow gene synthesis and lentiviral delivery of the corresponding effectors. Sequence diversification was achieved by (i) optimization of the codon usage to maximize expression in mammalian systems using a tool available online (Integrated DNA technology, IDT), (ii) introducing amino acid changes at positions 4, 11, 24, and 32, which are polymorphic in natural TALE-DBD of *Xanthomonas* bacteria [8], and (iii) manual optimization of the remaining repetitive DNA sequences that are longer than eight base pairs where possible. This resulted in a reduction of inter-repeat DNA sequence identity from an average of 93.9% to 77.4% (Supplementary Figure S2). Next, to further increase the diversity of inter- and intra-repeat DNA sequence, we embedded repeat modules from TALE-like proteins found in the bacterium *Burkholderia rhizoxinica* within the sequence optimized TALE-DBD. These proteins are characterized by repeat units having considerably different amino acid compositions as compared to those of *Xanthomonas* TALEs, despite retaining similar DNA recognition properties [19]. Since we anticipated that co-existence of repeat modules from different bacterial TALEs might destabilize the DNA binding, we substituted the *Xanthomonas* repeat modules stepwise. First, we investigated whether the position of one *Burkholderia* module could affect the stability and functionality of the resulting effector. Thereto we introduced a single *Burkholderia* module at three different positions of the optimized DNA binding domain. To increase the chimerization level of the TALE-DBD we included up to five *Burkholderia* modules for a total of six architectures with increasing levels of sequence variation (Figure 1a).

This multistep chimerization strategy was used to re-assemble the left arm (L-98) of a previously published TALE nuclease (TALEN) pair targeting the human *CCR5* gene (TC06) [6]. To assess whether the different steps of chimerization impact on the resulting protein stability, we transfected HEK293T cells using plasmid DNA encoding for the canonical (L-98) or for either of the chimerized TALEN L-98. Steady-state expression levels, 48 h after transfection, showed that all the chimerized TALENs tested were expressed at similar levels (Figure 1b, left panel). The effector harboring five *Burkholderia* modules showed a slightly lower expression level (Figure 1b, right panel). To monitor whether the optimization strategy resulted in a reduction of the TALEN activity, we combined either the canonical or the chimerized L-98 TALENs with the corresponding right arm of the TC06 nuclease pair (i.e., R-101) and transfected HEK293T cells with the matching expression plasmids. Three days later, we measured the extent of NHEJ-mediated mutagenic repair at the *CCR5* target site using the mismatch-sensitive T7 endonuclease 1 (T7E1) assay, as previously published [6]. All but one nuclease pair showed similar cleavage activity suggesting that, while the incorporation of three *Burkholderia* modules only slightly reduced the corresponding TALEN cleavage activity, five modules completely abolished it (Figure 1c).

Having defined that *Xanthomonas* TALE arrays tolerate up to three *Burkholderia* modules in positions 2, 8, and 15 resulting in an average DNA sequence identity to canonical DBD of 70.7% (Supplementary Figure S2), we used this scaffold in the subsequent experiments. To substantiate that chimerized TALEs are as effective as their canonical counterpart in different effector contexts, we chimerized a previously published TALE-based DNA binding domain targeted to intron 1 of the *CXCR4* gene [4] and ordered it in two gene synthesized fragments. The chimerized DBDs were used to generate transcriptional activators by fusion to a VP64 transcriptional activation (TA) domain linked to a green fluorescent protein via a T2A self-cleaving peptide. This resulted in the generation of *CCR5*- and *CXCR4*-specific activators (Figure 1d). To test the activity of the chimerized TAs, we generated a reporter construct harboring a mCherry coding sequence with an upstream minimal promoter fragment from the HSV thymidine kinase (TK) and tandem repeats of either *CCR5*- and *CXCR4*-specific target sites. To normalize for transfection efficiency, the reporter construct contains an enhanced blue fluorescent protein (EBFP) expression cassette (Figure 1d). The ability of the canonical or chimerized TALE-TAs to activate mCherry expression was monitored 48 h after cotransfection of the reporter construct and the respective TA expression vector. TAs harboring either a canonical or a chimerized DNA binding domain were similarly effective and promoted mCherry expression two- or three-fold over background, respectively (Figure 1d and Supplementary Figure S3).

### 2.2. Chimerization of TALE DNA Binding Domains Enables Lentiviral Delivery

**Figure 2:**
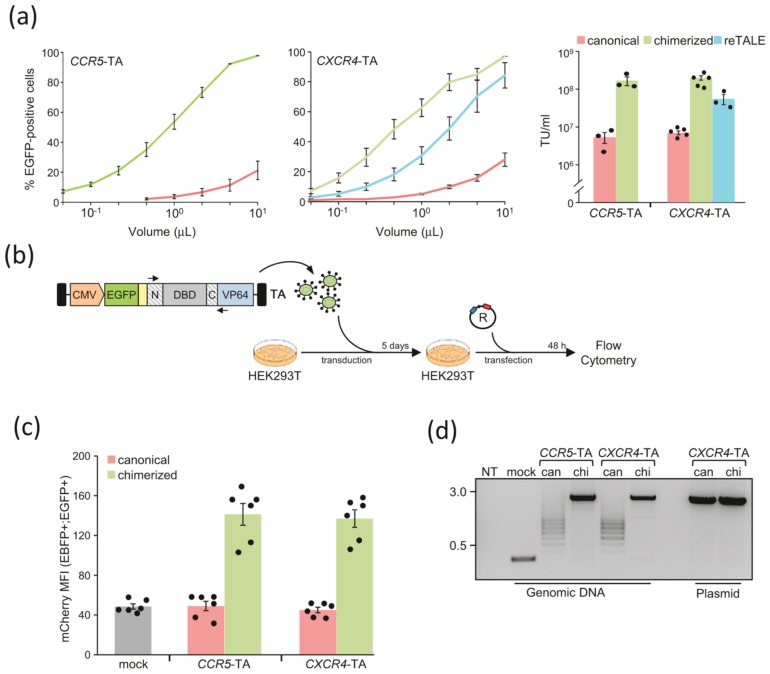
Lentiviral delivery of chimeric TAs: (**a**) Titration of lentiviral vectors expressing canonical, chimerized, or recoded TAs (reTALE). Increasing amounts of lentiviral vectors expressing either canonical (light red), chimerized (light green), or recoded (light blue) TAs targeting *CCR5* or *CXCR4* genes, respectively, were used to transduce HEK293T cells. The extent of EGFP positive cells was measured by flow cytometry and used to determine the viral titer as transducing unit (TU)/mL (right; mean ± SEM). Each dot represents a single data point. (**b**) Experimental design. HEK293T cells are transduced with the lentiviral vectors containing the indicated *CCR5*- or the *CXCR4*-specific TAs. Five days later, transduced cells are transfected with the reporter plasmid (R), described in Figure 1d, and mCherry expression levels measured via flow cytometry two days later are used to estimate TA activity. (**c**) Activity of TAs. The graph indicates the extent of mCherry expression (mean ± SEM), measured as mean fluorescence intensity (MFI), in the fraction of cells that received the reporter plasmid (EBFP+) and stably express the TA as a consequence of lentiviral vector integration (EGFP+). Basal mCherry expression levels are measured by using a lentiviral vector containing an effector lacking the DNA binding domain (mock, grey bar). Each dot represents a single data point. (**d**) Analysis of TAs integrity. Seven days post transduction, cells were harvested, and genomic DNA was purified to assess the integrity of the TA expressed from integrated vectors. The integrity of the DNA binding domain (DBD) included in the respective TA is assessed via PCR amplification using the primers depicted in panel b (black arrows). As a positive control, the same PCR is performed on the corresponding plasmid DNA to visualize the size of the amplicon derived from a full-length DBD. DNA marker (in kbp) is indicated on the left.

We next explored the possibility to use lentiviral vectors (LV) to deliver effectors containing a chimerized TALE-DBD. We generated LVs containing *CCR5*- or *CXCR4*-specific TA and used these vectors to transduce HEK293T cells. We first measured the titer of the LV produced via flow cytometry taking advantage of an enhanced green fluorescent protein (EGFP) expression cassette contained in the TA vector (Figure 1d). Increasing the dose of the different LV used to transduce the cells led to increasing amount of EGFP+ cells (Figure 2a, left panels). Using lentiviral vectors harboring chimerized TALE-TA resulted in markedly higher percentage of EGFP-positive cells and to an average increase of the vector titers of about 10- to 15-fold (Figure 2a, right panel and Supplementary Figure S4). Previous efforts have shown that reducing the repetitiveness of the DNA sequence coding for a TALE-DBD allow lentiviral based delivery of TALE-nucleases [18]. Interestingly, on the one hand, lentiviral vectors containing *CXCR4*-specific TA harboring either a recoded or a chimerized TALE-DBD showed comparable titers (Figure 2a). On the other hand, transducing the cells with a lentiviral vector harboring only a partially chimerized DBD resulted in low EGFP expression, suggesting that codon variation alone is not sufficient to allow reverse transcription of the complete vector genome during transduction (Supplementary Figure S5). To test whether the vectors also encode active TALE-TAs, we first transduced HEK293T cells with the LV vectors containing the *CCR5*- or *CXCR4*-specific TAs and five days later we transfected these cells with the mCherry reporter construct described above (Figure 1d). Using flow cytometry, we measured the activity of the TALE-TA by calculating the expression levels of mCherry in the subpopulation of cells that express both the vector and the reporter construct (EGFP+ and EBFP+). Interestingly, cells transduced with LV containing the chimerized TALE-TAs showed up to three-fold mCherry activation as compared with controls. In contrast, activation of reporter gene expression failed in cells receiving LV containing the canonical TALE-TAs (Figure 2c). To confirm that chimerization of TALE-based DNA binding domain enabled the lentiviral delivery of full-length TALE-TAs, we extracted genomic DNA from cells transduced with these LVs and PCR amplified the TALE domains. As expected, cells transduced with a LV containing a TALE-TA lacking the DBD (i.e., mock) resulted in a small amplification product of the expected size indicative of proper vector integration. However, no full-length viral genome could be retrieved in genomic DNA extracted from cells transduced with LV coding for canonical TALE-TAs. The presence of shorter amplification products suggests that rearrangements have occurred during the reverse transcription of the viral genome, as previously shown [16,17]. In contrast, analysis of DNA from cells transduced with LV encoding chimerized TALE-TA reveals a single amplification product of the expected length (Figure 2d), supporting the notion of error-free viral genome replication and integration.

**Figure 3:**
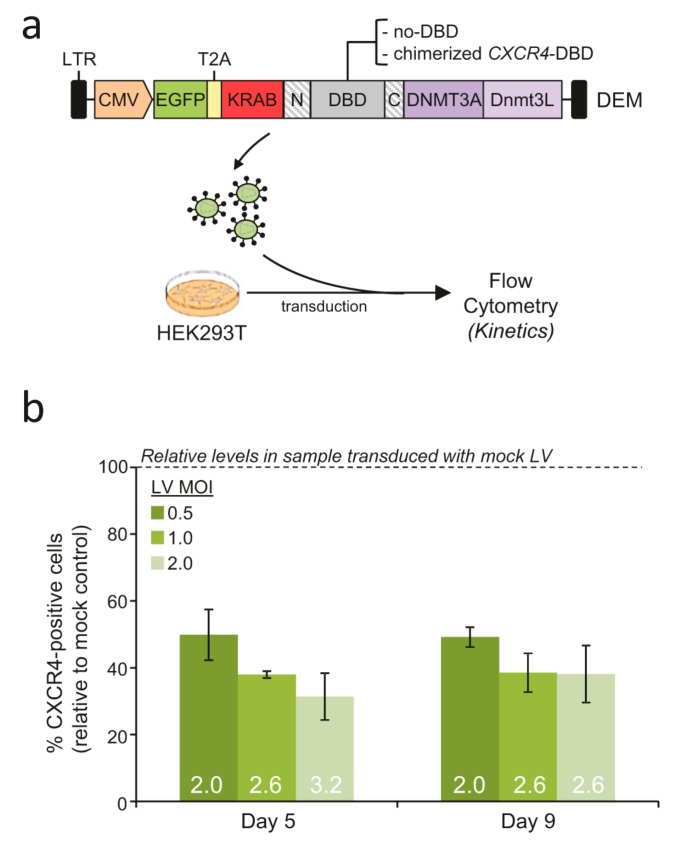
Lentiviral delivery of chimerized designer epigenome modifiers (DEMs). (**a**) Schematic of the designer epigenome modifiers harboring a chimerized TALE DNA binding domain. Lentiviral vectors containing either an active or inactive (lacking the DNA binding domain) DEM were generated and used to transduce HEK293T cells. (**b**) Activity of chimerized DEM. Cells were transduced with increasing doses of the lentiviral vectors described in a and CXCR4 expression was measured at different time points after transduction via flow cytometry. The histogram shows the extent of CXCR4+ cells (mean ± SEM) in samples transduced with lentiviral vectors encoding for an active DEM relative to those receiving the mock vector (containing a non-targeted DEM lacking the DNA binding domain). Fold change values relative to samples transduced with the mock LV are reported within the bar graph.

We next investigated whether chimerized TALE domains could be combined with the designer epigenome modifier (DEM) platform that we described recently [4]. To this end, we chimerized a previously described *CXCR4*-specific DEM and used a non-targeted DEM (lacking the DBD) as a negative control (Figure 3a). We generated lentiviral vectors and transduced HEK293T cells with increasing amounts of LV containing either the chimerized *CXCR4*-specific DEM or the non-targeted control. CXCR4 protein levels were measured via flow cytometry five, and nine days, post transduction. CXCR4 positive cells were markedly reduced in cells transduced with the LV containing the chimerized DEM from 30% to 8% on average (Supplementary Figure S6). This resulted in a up to three-fold decrease in the ratio of CXCR4-positive cells in samples receiving the highest LV dose as compared with cells transduced with the control vector (Figure 3b and Supplementary Figure S6). These results suggest that chimerization of the TALE-based DNA binding domain allows for the delivery of full-length designer epigenome modifiers that retain their activity.

## 3. Discussion

The availability of various DNA targeting platforms has allowed researchers to direct the activity of designer effectors to precise genomic locations. DBDs derived from eukaryotic zinc finger (ZF) proteins or from bacterial TALE or CRISPR-Cas systems have been engineered to specifically recognize defined DNA sequences in the genome of choice. This has prompted the generation of designer nucleases, artificial transcription factors, and more recently, epigenome modifiers which have been largely employed to achieve targeted and precise changes in the genome, transcriptome, or epigenome of a cell, respectively. Given the growing interest in pursuing targeted cellular modifications, it is crucial to expand the delivery tools available for these customized effectors. Most genome editing strategies rely on a so-called hit-and-run approach that does not necessitate sustained expression of the effector moiety. In contrast, transcriptome and epigenome editing could require the continued expression of the chosen effectors in some conditions. Integrating LVs are effective tools for achieving persistent transgene expression which have been widely used in a variety of applications spanning from basic research to human gene therapy [20]. However, despite their flexibility, so far, LVs have failed to deliver intact effectors containing a TALE-based DBD due to the highly repetitive nature of these structures that impairs viral genome integrity during the replication of recombinant vector genomes [16,17].

To overcome this limitation and expand the applicability of customized effectors containing a TALE-DBD, we have devised a strategy to drastically reduce the repetitiveness of DNA sequences encoding for TALE-based DNA binding domains. Similar to previous reports [18], we have heavily varied the codon usage of the DNA sequence encoding for TALE-DBD combining both a codon usage optimization and manual alteration of repetitive DNA sequences longer than eight base pairs. To further increase sequence diversity, we introduced amino acid changes at naturally polymorphic positions of *Xanthomonas* TALEs [8] and chimerized the new DBD by substituting three repeat modules with equivalent repeats from TALE proteins found in the bacterium *Burkholderia rhizoxinica*. These represent an ideal candidate to increase sequence diversity of a TALE-DBD as they are typically shorter and show a highly diverse amino acid sequence as compared with *Xanthomonas* TALEs, despite retaining their same DNA recognition code [19]. We show that the incorporation of *Burkholderia* modules is limited to three, as a DBD harboring five foreign units completely abolishes the activity of chimerized designer nucleases. This suggests that structural stability of the DBD could be altered by the addition of foreign TALE modules, probably due to the different length and amino acid composition of the *Burkholderia* modules. This in turn might either directly affect DNA binding or alter the right-handed superhelix of the TALE-DBD, resulting in misalignment between the C-terminal RVDs and their corresponding target base pairs in the DNA. In this context, specificity of chimerized effectors could be altered and therefore it is important, in the future, to assess the impact of chimerization on tolerance of the DBD for off target binding. Indeed, a proper side-by-side comparison of effectors harboring either a canonical or a chimerized TALE-based DNA binding domain is crucial to highlight changes in off targeting effects that could be due to the novel chimerized structure.

Having established the architecture resulting in functional chimerized effectors, we explored the possibility of using lentiviral vectors for their delivery into target cells. We have previously shown that the generation of recombinant LVs containing TALE-based nucleases results in major viral genome rearrangements encompassing the region of the TALE-DBD during viral replication [16,17]. This eventually leads to the delivery of aberrant nucleases which could still exert their functions at unintended genomic sites bound by the remaining of the rearranged DBD. In this study, we show that, when chimerized, the DNA sequence encoding for TALEs is tolerated during the generation of recombinant lentiviral vectors and results in high titers of LV vectors containing previously described TALE-based transcription factors and designer epigenome modifiers [4,6] which retain their activity.

Previous strategies have relied exclusively on computational evolution of TALE-DBD coding sequence to minimize repetitiveness and enable the delivery of so-called “recoded TALEs” (reTALEs) using lentiviral vectors [18]. In a side-by-side comparison we showed that LVs containing either chimerized or recoded TALEs targeting *CXCR4* have comparable titers confirming that reTALE sequences are tolerated during viral replication. However, reTALE-DBDs still retain sequence repeats up to 11 base pairs in length that prevent their generation by synthesis of small gene fragments which typically tolerates only repeats shorter than eight nucleotides. This limits the generation of customized reTALE-based DNA binding domains that still relies on complex PCR-based amplification strategies, as reported in [18], or to costly synthesis of complex genes. Our chimerization strategy overcomes this limitation as customized DBDs can be simply purchased from a gene synthesis company as two gene fragments (Supplementary Figure S1). The addition of suitable overlapping ends enables the assembly of the DBD in one step via Gibson Assembly directly in the final destination vector containing the desired effector domain. This feature should allow any laboratory to approach the TALE-based designer effector technology, therefore, fostering the widespread use of this technology to similar levels as observed when RNA-guided effectors based on the clustered regularly interspaced short palindromic repeat/CRISPR-associated (CRISPR/Cas) system have been introduced [21]. The two components nature of this platform requires that the expression of the effector protein (i.e., the Cas9 or the dCas9 fusion) and the small RNA molecule used for the genomic targeting (i.e., the guide RNA) is fine-tuned to maximize the intracellular assembly of functional CRISPR-Cas effectors. Moreover, the targeting range of this system is limited by the PAM requirement of the Cas proteins [22]. TALE-based effectors have a higher targeting range as they only require a 5′T for binding [8] and their single-component nature facilitates intra-cellular availability. Having a new delivery vehicle for designer effectors containing a TALE-derived DNA binding domain could certainly expand the potential use of these moieties for in vivo applications in animal models to increase our knowledge of basic biological mechanisms but also to test innovative potential therapeutics.

## 4. Materials and Methods

### 4.1. Plasmids Construction

Canonical TALE-based DNA binding domain were engineered using Golden Gate cloning, as previously described [23]. Repeat modules with codon varied DNA sequences as explained in the text or derived from *Burkholderia rhizoxinica* TALE-like proteins were chemically synthesized as gene fragments at Integrated DNA Technology (IDT). The gene fragments were cloned via Gibson Assembly and introduced within the proper Level 1 module vectors digested with BsaI and retaining the same overhangs for the subsequent Golden Gate cloning steps [23]. Chimerized TALE-based DNA binding domain targeting the *CCR5* gene coding region (optL-98 and derivatives) were generated following the same procedure as for canonical DBDs via Golden Gate cloning, as previously published [23], but using the newly generated Level 1 vectors. The DNA binding domains were then cloned into our previously optimized TALE-nuclease scaffold (∆135/+17) [24] containing the wild type *Fok*I endonuclease domain to generate canonical and chimerized designer nucleases. Subsequently, the *Fok*I domain was substituted with a VP64 activator and the whole coding sequence of the effectors was introduced in the multiple cloning site (MCS) of a modified pCCL_CMV-EGFP-T2A-MCS vector containing an enhanced green fluorescent protein (EGFP) expression cassette driven by a cytomegalovirus (CMV) promoter followed by a T2A self-cleaving peptide resulting in the pCCL_CMV-EGFP-T2A-TA. In this context, the effector is driven by the CMV promoter in a monocistrionic mRNA containing both the EGFP and the effector which is released upon translation. The chimerized TALE-based DNA binding domain targeting the intron 1 of the *CXCR4* gene (optR2) was purchased as two gene fragments of 882 and 1′172 base pairs, respectively, at Integrated DNA Technology (IDT; Supplementary Figure S1) while the corresponding reTALE DBD was ordered as full gene synthesis product at the same company. The gene fragments contained overhangs that permit the assembly of the chimerized DBD directly in the final destination vector either containing the VP64 transcription activator domain (Figure 1d) or the epigenome modifier scaffold previously optimized (Figure 3a) [4] linearized with *Xho*I and *BamH*I. To generate canonical or chimerized designer epigenome modifiers (DEMs), the DEM coding sequence was introduced in the pCCL_CMV-EGFP-T2A-TA using NheI and BamHI restriction enzymes, resulting in the pCCL_CMV-EGFP-T2A-DEM. To generate the mCherry reporter construct, the pCCL_CMV-EGFP_T2A_MCS, as described above, was linearized with *Xho*I and *BstB*I and ligated via Gibson Assembly to a gene fragment (IDT) containing the effector target sites in tandem followed by a TATA-box, the mCherry coding sequence, and an SV40 polyadenylation signal. Subsequently, the EGFP coding sequence was released using *BspE*I and *BsiW*I and substituted with the coding sequence of an enhanced blue fluorescence protein (EBFP) amplified via PCR from the 2106_pEBFP-C1 plasmid. All the plasmid maps and sequence information are available upon request to the corresponding author of this manuscript.

### 4.2. Reporter Assay in HEK293T Cell Line

HEK293T cells were maintained in a humidified incubator at 37 °C and 5% CO2 in Dulbecco’s modified Eagle medium (DMEM; Gibco, Life Technologies, Waltham, MA, USA) supplemented with 10% fetal calf serum (FCS; PAA, Pasching, Austria), 1% penicillin/streptomycin (Sigma-Aldrich, St. Louis, MO, USA), and 1% sodium pyruvate (Biochrom, Berlin, Germany). To perform the reporter assay, 10^5^ HEK293T cells were seeded into an adherent 24-well plate in 500 µL medium. After 24 h, cells were transfected in duplicate using polyethylenimin (PEI) with a transfection mix containing 200 ng of effector plasmid (pCCL_CMV-EGFP-T2A-TA) and 25 ng of mCherry reporter plasmid. The total amount of DNA was kept constant by adding pUC118 to 1.2 μg. Eight hours later, 300 µL medium for each well was replaced with fresh medium and cells were harvested 48 h after transfection in FACS buffer (100 mM EDTA, 0.1% Na-Azide, 5% FCS prepared in PBS). Fortessa flow cytometer (BD Biosciences, San Jose, CA, USA) was used to measure the extent of mCherry, enhanced green fluorescent protein (EGFP) and enhanced blue fluorescent protein (EBFP) signals 48 h post transfection, and data were analyzed using FlowJo v.10 (FlowJo LLC).

### 4.3. Lentiviral Vectors Production

To generate lentiviral vectors pseudotyped with vesicular stomatitis virus glycoprotein-G (VSV-G) containing the described effectors, 1.5 × 10^7^ HEK293T cells were seeded in a 15 cm dish with 25 mL of medium. After 24 h, cells were transfected with a mix containing 31 µg Gag/Pol, 13 µg Rev, and 3.7 µg VSV-G plasmids, in addition to 19 µg of effector expression plasmid, either transcription activator (TA) or designer epigenome modifier (DEM) described within the main text. The volume of the DNA mixture was adjusted to 1250 µL using 150 mM NaCl. Subsequently, 1250 µL of PEI were added to the DNA mix, and after, 10 min incubation, the solution was distributed dropwise to the cells. After 16 h, the medium was removed, and the cells were overlaid with 15 mL fresh medium supplemented with 10 mM sodium butyrate. Supernatants were harvested 40 h and 64 h after transfection and filtered using 0.45 µm filters. To concentrate the viral solutions, ultracentrifugation was carried out using Sorvall WX 80 Ultra Series (Thermo Fisher Scientific, Waltham, MA, USA) ultracentrifuge. In brief, 36 mL filtered supernatant were overlaid on 4 mL of cold 20% sucrose solution and centrifugation was conducted at 117,372.3 RCF at 4 °C for 2 h. Then, the pellets containing the viral particles were resuspended using 20 to 40 µL cold PBS. Vector titers were determined through limiting dilutions on HEK293T cells seeded at a density of 5.0 × 10^4^ cells per well in 48-well plates. Three days post transduction, frequencies of EGFP-positive cells, resulting from the EGFP expression cassette present in the effector expression plasmid (Figure 1d) were determined through flow cytometry by using a BD Accuri flow cytometer (BD Biosciences, San Jose, CA, USA). As a result, the titers are expressed in terms of transducing units per milliliter (TU/mL).

### 4.4. Structural Analysis of Integrated Lentiviral Vectors and Activity of Corresponding TALEs

HEK293T cells were transduced with lentiviral vectors containing the different effectors described at a multiplicity of infection (MOI) of 0.2. Four days post transduction, 10^5^ transduced cells were seeded in a 24-well plate for functional analysis of the delivered effector while the remaining cells were seeded in 12-well plates for structural analysis of the integrated lentiviral vector. To assess the functionality of the effectors upon lentiviral delivery, 24 h after seeding, cells were transfected with a mix containing 25 ng of mCherry reporter plasmid using PEI. The total amount of DNA was kept constant by adding pUC118 to 1.2 μg. Cells were harvested 48 h after transfection in FACS buffer and mCherry, EGFP and EBFP fluorescent signals were measured using a Fortessa flow cytometer (BD Biosciences, Allschwil, Switzerland), as described above. For structural analysis of the integrated viral genome, cells were harvested seven days post transduction and the genomic DNA was isolated using QIAamp DNA Blood Mini Kit (Qiagen, Hilden, Germany). The integrity of the TALE-TA DNA binding domain was assessed via PCR amplification using the forward primer 5′-gccgtggaagccgtgc-3′ and the reverse primer 5′-ccaggtcaaagtcatcgagggc-3′. As a positive control, amplification was performed using the corresponding plasmid DNA as template to visualize the size of the amplicon derived from a full-length DBD.

### 4.5. Analysis of DEM Activity

HEK293T cells were transduced with lentiviral vectors expressing a designer epigenome modifier (DEM) containing a chimerized DBD targeted to the *CXCR4* gene. A lentiviral vector containing a DEM lacking the DBD was used as mock control. Cells were transduced at a MOI of 0.5, 1, and 2, respectively. At different days after transduction the cells were harvested and stained for CXCR4 expression using anti-human CD184-APC (BD, 306510). CXCR4 expression levels were measured in the fraction of transduced cells (EGFP+) using a BD Accuri flow cytometer (BD Biosciences, San Jose, CA, USA). The histogram in Figure 3b shows the amount of CXCR4 positive cells in the samples receiving different doses of vector containing the chimerized DEM relative to the amount of CXCR4 positive cells in samples transduced with the mock vector.

## Supplementary Materials

Supplementary materials can be found at https://www.mdpi.com/1422-0067/21/3/795/s1.

## Abbreviations

TALE	Transcription activator-like effector
DEM	Designer epigenome modifier
TA	Transcriptional activator
DBD	DNA binding domain
