# Supplementary material for: Chimerization Enables Gene Synthesis and Lentiviral Delivery of Customizable TALE-Based Effectors
Source: Int J Mol Sci. 2020 Jan 25;21(3):795. doi: 10.3390/ijms21030795 (PMC7038218; doi:10.3390/ijms21030795)
Supplement: Supplementary file 1 [file ijms-21-00795-s001.pdf]

Supplementary Figure S1

Fragment A (882 bp)

gtggtccggcgctagagcactcgaggccttgctgacagtggccggcgagctgagaggccctccactgcagctggacaccggccagctgctgaagatcg
ccaagcggggaggcgtgacagccgtggaagccgtgcacgcttggcggaatgccctgacaggcgctcccctgaaccttacgccagaccaggtcgttgc
catcgcaagcnnnnnnggaggaaaacaggctctggagacagtccagagacttcttccggtgttgtgccaggaccacggtttttctcaggccgacatc
gtgaaaatcgccggtnnnnnnggtggcgcacaggccctacaggcggtactggaattggaacccaccctgcgcgaaaggggactcacaccacaaca
ggtggtcgccatagctnnnnnnattggaggaaagcaggctcttgagactgtgcagcgcctgctcccagttctgtgtcaggctcatggcctcaccccag
atcaggtggttgccatcgcttctnnnnnngggggcaaacaagccctggagactgtccaacggctgctcccggttttgtgccaggatcacggccttacc
ccagcacaggtcgttgccattgcatccnnnnnnggcggtaaacaggctctggaaactgtccaagcgttgctgcctgtactctgtcaggaccacggatt
gacccctgaccaagtcgttgcaatcgcaaatnnnnnnggcgggaagcaggctctggaaactgttcaaagactccttcccgttctctgtcaggcacacg
gactgaccccagaacaggtcgtggcaatcgcttcannnnnnggaggcaaacaggctctcgaaacagtgcagaggctccttccggtcctgtgtcaggc
ccacgg

Fragment B (1172 bp)

ggtcctgtgtcaggcccacggattctcacaggcagacatcgtgaagattgctggcnnnnnnggtggcgcgcaagcactccaagcggtgttggaattg
gagcccactctccgggaaagggggctcacgcccgatcaagtagtggcaattgcaagtnnnnnnggaggaaaacaagctcttgaaaccgtgcaaag
actccttccggtgttgtgtcaagatcatggcctgacgccggcccaagttgtggccattgcttctnnnnnnggggtaagcaagcccttgaaactgtcca
ggccttgctgcccgtgctgtgtcaagctcacggactcacaccagaccaagtggttgccattgcaaatnnnnnnggaggaaaacaagcgttggagact
gttcaacgattgcttccagtgctttgtcaagcccatggtctgacacctgagcaagtagtggcaatagcatctnnnnnnggggtaaacaggcccttga
gactgtacagcgccttttgcctgtcctgtgtcaagaccatgggctgacgcccgagcaggtagtagccatagcttctnnnnnnggcggaaagcaagca
ctggagaccgtccagcgcctccttcctgtactctgccaagcgcacgggttgacgcctcaacaagtagtggcaatcgcgaacnnnnnnggaggcaaac
aggctctggagacagttcaaaggcttctcccagtgttgtgtcaagcacacggttactccagagcggatattgtcaaaattgccggannnnnnggtggg
gcgcaggcccttaaagccgtagtgatgcacggacctacgctttgcgaacgcggactcacgccggatcaggttgttgccatagctaatnnnnnngggg
ggaaacaagctttggaaacagttcaaagacttcttcctgtgctctgtcaagaccatggactcaccccagagcaagttgtcgcaattgcatcannnnnn
ggtggtaagcaggccctagagaccgtacagcgcttgctccctgtcctttgccaggaccacggactcactccgcaacaagtggtcgcaatcgcctctnn
nnnnggaggacgacctgcgcttgaatctattgttgcccaattgtcccgaccggaccccgctctggcagctctcacaggatccgggcgcgccgacgcgc
tgg

Supplemenatry Figure S1. DNA sequence of chimerized TALE-based DNA binding domain (DBD). Chimerized TALE-DBD can be generated via gene synthesis as two gene fragments (indicated as A and B) with compatible overhangs (underlined) necessary for Gibson assembly. For one step cloning into the final destination vector containing our previously optimized designer nuclease, transcription factor or epigenome modifier scaffolds [4, 6], the vectors can be linearized with XhoI and BamHI resulting in overhangs compatible with those of the two fragments above (double underlined). The multiple "n" indicate the position of the hypervariable basepairs coding for the repeat variable di-residues (RVDs) that can be customized to generate chimerized DBDs targeting the sequence of choice.

Supplementary Figure S2

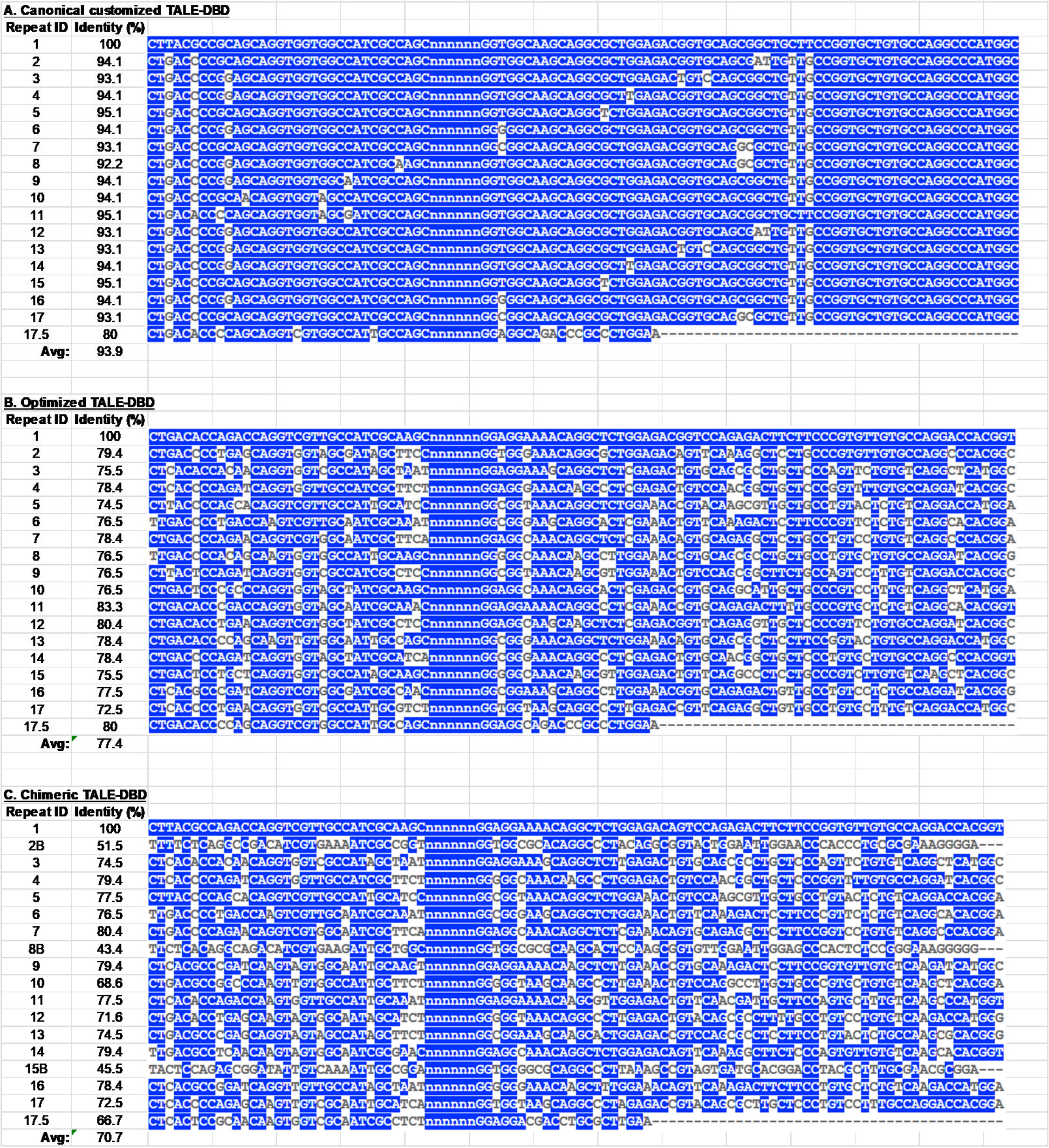

Supplementary Figure S2. Results of TALE-based DNA binding domain chimerization. The figure shows the inter-repeat DNA sequence identity for canonical (**A**), optimized (**B**), or chimerized (**C**) TALE-based DNA binding domains. The blue color indicates base pairs identical to the corresponding in the first repeat taken as a reference. The position of the six "n" indicates the hypervariable base pairs coding for the repeat variable di-residues (RVDs).

Supplementary Figure S3

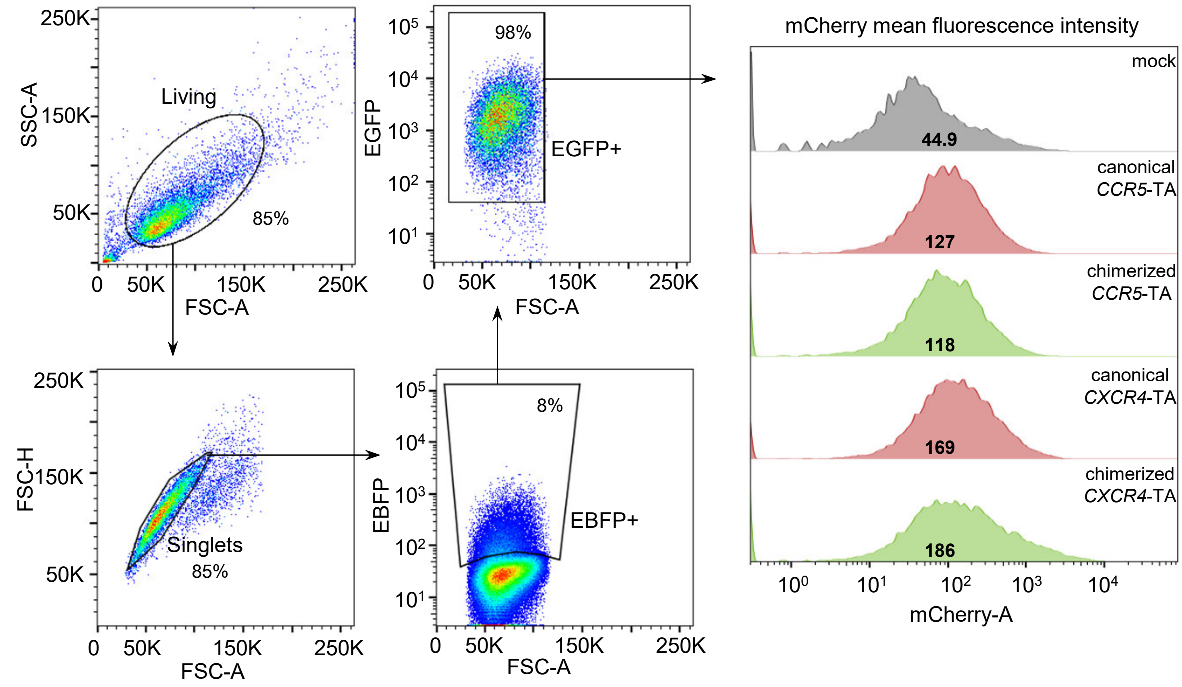

Supplementary figure S3. Functionality of chimerized TALE-based transcriptional activators. TALE-TA expression plasmids containing either the canonical (light red) or the best-performing chimerized (light green) DNA binding domain were co-transfected with the reporter construct described (Figure 1d) and the ability of the indicated TA to drive mCherry expression was assessed via flow cytometry, 48-hours post transfection. The gating strategy (left) and the results obtained for a representative experiment (right) are indicated. The mCherry expression levels are measured as mean fluorescence intensity (MFI), in the fraction of cells that received both the effector and the reporter plasmids (EGFP+ and EBFP+ respectively) and is indicated in bold numbers in the right panel. Basal mCherry expression levels are measured by transfecting an effector plasmid lacking the DNA binding domain (mock, grey).

Supplementary Figure S4

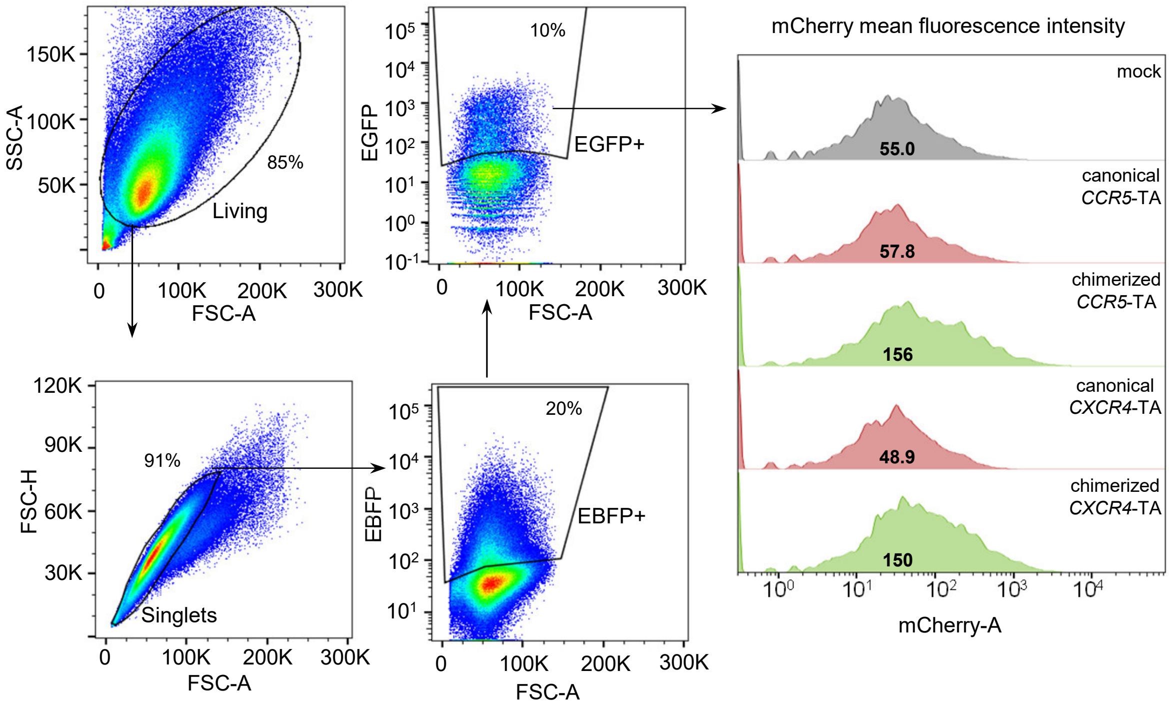

Supplementary figure S4. Lentiviral delivery of chimerized TALE-based transcriptional activators. HEK293T cells were transduced with lentiviral vectors containing the different effectors indicated at a multiplicity of infection (MOI) of 0.2. Five days later, the transduced cells were transfected with the reporter plasmid shown in Figure 1d. The gating strategy (left) and the results obtained for a representative experiment (right) are indicated. The mCherry expression levels are measured as mean fluorescence intensity (MFI), in the fraction of cells that received both the effector and the reporter (EGFP+ and EBFP+ respectively) and is indicated in bold numbers in the right panel. Basal mCherry expression levels are measured in cells transduced with a lentiviral vector containing an effector lacking the DNA binding domain (mock, grey).

Supplementary Figure S5

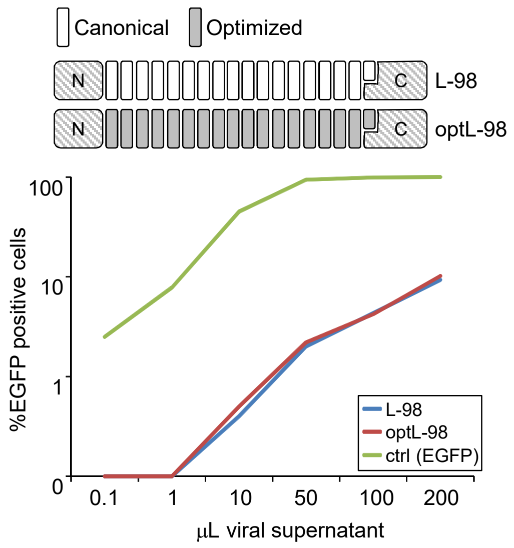

Supplemenatry Figure S5. Codon variation is not sufficient to enable the generation of lentiviral vectors containing TALE-based effectors. The graph shows the amount of enhanced green fluorescent protein (EGFP) positive cells resulting from transducing HEK293T cells with increasing amounts of the indicated recombinant lentiviral preparation containing the canonical (L-98) or optimized (optL-98) TALE nucleases fused to the EGFP via T2A peptide. A vector encoding for EGFP is used as control (ctrl, EGFP).

Supplementary Figure S6

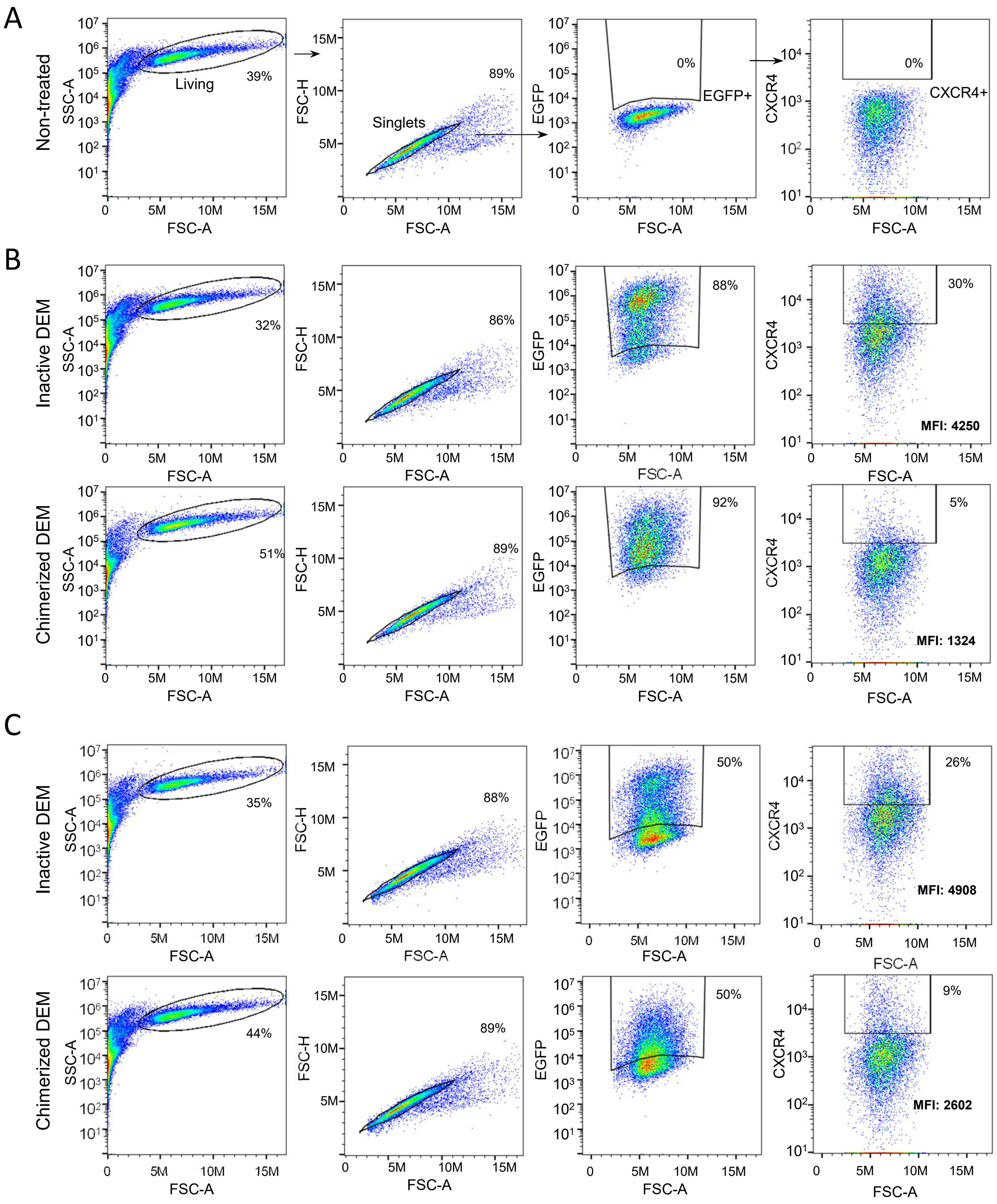

Supplementary Figure S6. Lentiviral delivery of designer epigenome modifiers (DEMs). HEK293T cells were transduced with a lentiviral vector containing either a chimerized or inactive (lacking the DNA binding domain) DEM at a multiplicity of infection (MOI) of 0.2. The figure describes the gating strategy setup in non-treated cells (**A**) and the results obtained for a representative experiment in which CXCR4 expression levels are measured at day 5 (**B**) or 9 (**C**) post transduction. The mean fluorescence intensity (MFI) of CXCR4 expression in the area highlighted is indicated in the last panel.
